# Multiomic Analysis of Environmental Effects and Nitrogen Use Efficiency of Two Potato Varieties Under High Nitrogen Conditions

**DOI:** 10.3390/plants14050633

**Published:** 2025-02-20

**Authors:** Shuo Gao, Guowei Wei, Yuan Chang, Yanbin Yin, Qiaorong Wei, Ying Shi

**Affiliations:** 1 College of Agriculture, Northeast Agricultural University, Harbin 150030, China; 15146011604@163.com (S.G.); 13349391930@163.com (G.W.); yuan29291314@163.com (Y.C.); yybnice@neau.edu.cn (Y.Y.); 2 National Key Laboratory of Smart Farm Technologies and Systems, Harbin 150030, China; 3 Key Laboratory of Germplasm Enhancement, Physiology and Ecology of Food Crops in Cold Region, Ministry of Education, Harbin 150030, China

**Keywords:** rhizosphere microorganisms, nitrogen use efficiency, potato breeding, greenhouse gas

## Abstract

Potato (*Solanum tuberosum* L.) has high nutritional value and strong adaptability and plays an extremely important role in global food security. Excessive use of nitrogen (N) fertilizer in potato production has increased costs and environmental pollution. In this study, the N use efficiency (NUE) of two potato varieties (DXY and DN310) was determined under high nitrogen conditions. The N use efficiency of DXY was relatively high. The differences between the rhizosphere microbial population groups of the two varieties were determined using the metagenomic sequencing method. The genes related to N efficiency were jointly identified using transcriptome and metabolome analyses. Significant difference was observed between the two varieties of microorganisms, leading to different rhizosphere microorganisms. Compared with DN310, the roots of DXY retained more available N and generated less NO. Additionally, DXY exhibited relatively low disease susceptibility. Combined transcriptome and metabolome analyses indicated that the differentially expressed metabolites in the two different varieties under high N conditions were mainly enriched in amino acid metabolism and sugar metabolism pathways. Using weighted gene co-expression network analysis, two genes associated with N fertilizer response were identified: *PGSC0003DMG400025888* and *PGSC0003DMG400017276*. This study provided valuable insights into breeding potato varieties with high N efficiency.

## 1. Introduction

Currently, chemical fertilizers are being increasingly used in crop production. Chemical fertilizers can provide nitrogen, phosphorus, potassium, and other nutrients required for crop growth and development, among which nitrogen is the most important macronutrient element to drive plant growth and development [1]. For advanced plants, nitrate and ammonium are the main sources of nitrogen [2]. Soil microorganisms can oxidize ammonia nitrogen (NH_4_^+^-N) to nitrite (NO_2_⁻), which is further oxidized to nitrate (NO⁻). Nitrate can be further reduced to nitrogen gas (N_2_), thereby affecting the use of nitrogen in soil by plants [3]. The global use of nitrogen fertilizer has significantly increased over the past few decades. This has significantly impacted the environment, mainly in five aspects: soil acidification, water pollution, greenhouse gas emissions, decline in biodiversity, and soil salinization [4,5,6,7].

Subsurface root-soil-microbial interactions are extremely complex and critical to the growth, health, and adaptability of aboveground plants [8]. However, this interaction can also spread plant diseases, which has an impact on the growth and development of plants [9,10]. Plants exhibit two types of immunities: pattern-triggered immunity (PTI) and effector-triggered immunity (ETI). PTI is triggered by microbial patterns via cell surface-localized pattern-recognition receptors. To interfere with PTI, soil microorganisms secrete proteins called virulence factors, which may affect the structure and function of the soil microbial community [11]. This, in turn, affects the physical and chemical properties of the soil and plant growth [12,13]. Virulence-related genes encoding virulence factors can be classified into offensive, defensive, non-specific, and regulatory types according to their specific roles in the course of infection [14]. Offensive virulence factors help pathogens in invading the host, destroying host cells, obtaining nutrients, etc. Defensive factors can help pathogens escape the immune attack of the host to survive and reproduce in the host. Non-specific virulence factors are usually not specific to a specific pathogen but function as part of the host’s innate immune system. Regulatory virulence factors can adjust the virulence of the pathogen in response to environmental changes to better adapt to living conditions in the host. This classification helps to understand the specific roles of different virulence factors in the mechanism of pathogen infection. Analysis of pathogenic factors is helpful to study the disease resistance of plants under different microbiota conditions.

Potatoes play an extremely important role in global food security. Potato has high nutritional value. It is rich in proteins, vitamins, minerals, and other nutrients that are beneficial to humans [15]. Additionally, potato has strong adaptability and can maintain high yield stability under adverse conditions such as drought and high temperature [16]. However, potato has a shallow root system, which causes its water and nutrient requirements to be concentrated in the top layer of soil. Therefore, overapplication of nitrogen is common during potato cultivation. However, overapplication of nitrogen fertilizer leads to decrease in yield, quality deterioration, environmental pollution, increased production cost, and reduced nitrogen use efficiency [17,18,19]. Breeding varieties with high nitrogen efficiency is an effective way to improve the nitrogen use efficiency maintain high yield.

In this study, through field experiments using potato varieties with different nitrogen efficiency, rhizosphere microbial population changes under high nitrogen conditions were analyzed. Moreover, the effects of soil microorganisms on nitrogen utilization pathways of potato varieties with different nitrogen efficiency and virulence factors under high nitrogen conditions were studied. In addition, greenhouse simulation experiment was performed to compare gene transcription and metabolism levels in potato varieties with different nitrogen efficiency under high nitrogen conditions.

## 2. Results

### 2.1. Rhizosphere Microbial Community Composition of Two Potato Varieties with Different Nitrogen Efficiency Under High Nitrogen Conditions

**Figure 1:**
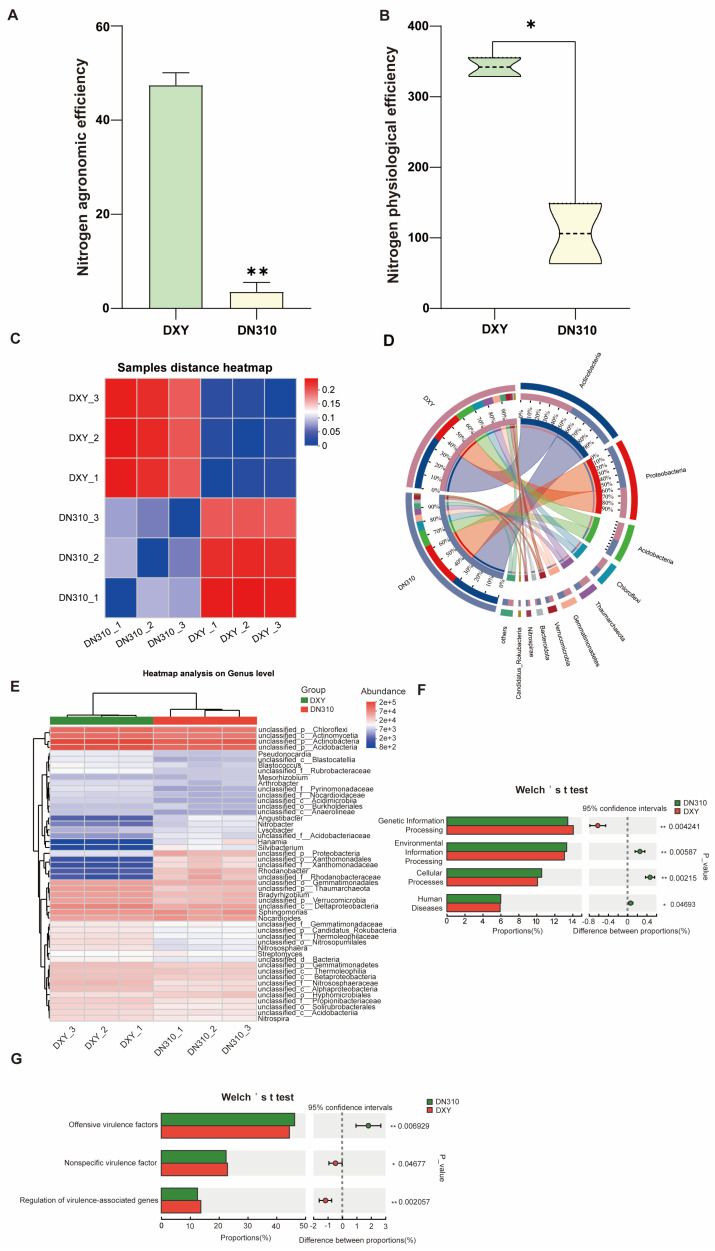
Differences in rhizosphere microorganisms of different potato varieties under high nitrogen conditions. (**A**) Comparison of nitrogen agronomic efficiency of DN310 and DXY under high nitrogen conditions. (**B**) Comparison of nitrogen physiological efficiency of DN310 and DXY under high nitrogen conditions. (**C**) Distance heatmap analysis of the sample. (**D**) Circos plot of the species–sample relationship. (**E**) Heatmaps of difference in rhizosphere soil microbial populations between DN310 and DXY under high nitrogen conditions. (**F**) KEGG pathway enrichment analysis of functions of rhizosphere soil microorganisms of DN310 and DXY under high nitrogen conditions. (**G**) Comparison of rhizosphere soil virulence factors of DN310 and DXY under high nitrogen conditions. (**F**,**G**) Welch’s test was used to evaluate the difference; * *p* < 0.05 and ** *p* < 0.01.

Nitrogen use efficiency is an important index of potato growth and development. The nitrogen agronomic efficiency and nitrogen physiological efficiency of the DXY variety was significantly higher than that of DN310 (Figure 1A,B). In general, compared with DN310, DXY had higher nitrogen use efficiency under high N conditions. To study the effects of high nitrogen on rhizosphere microorganisms of two potato varieties, the macrogenome of rhizosphere soil of DN310 and DXY was sequenced. First, the samples distance heatmap analysis of the sequencing results revealed that the distance between biological replicates of samples was small. The distance between samples of different varieties was large, indicating a good sampling efficiency (Figure 1C). The results of metagenomic sequencing revealed that there were 10 main bacterial groups in the two soil samples at the microbial phyla level: Actinobacteria, Proteobacteria, Acidobacteria, Chloroflexi, Thaumarchaeota, Gemmatimonadetes, Verrucomicrobia, Bacteroidota, Nitrospirae, and Candidatus_Rokubacteria. The relative abundance of Actinobacteria, Acidobacteria, Chloroflexi, Thaumarchaeota, Gemmatimonadetes, Verrucobacteria, and Nitrospira in the rhizosphere soil of DXY was 37%, 12%, 8%, 7%, 5%, 3%, and 2%, respectively, higher than that in the rhizosphere soil of DN310 (33%, 11%, 6%, 4%, 4%, 2%, and 1%, respectively). The relative abundance of Proteobacteria and Bacteroidetes in the rhizosphere soil of DXY was 21% and 1%, respectively, lower than that in the rhizosphere soil of DN310 (30% and 3%, respectively; Figure 1D). Metagenomic sequencing results revealed that the rhizosphere soil microbial populations of DXY and DN310 varieties were significantly different under high nitrogen conditions. The differences in the populations of unclassified_o_Xanthomonadales, unclassified_f_Xanthomonadaceae, Rhodanobacter, and unclassified_f_Rhodanobacteraceae were the most significant (Figure 1E). KEGG pathway enrichment analysis revealed that rhizosphere soil microorganisms in DXY were mainly enriched in environmental information processing, cellular processes, and human diseases under high nitrogen conditions, and those in DN310 were mainly enriched in genetic information processing (Figure 1F). The virulence factors indicating bacterial infectivity were analyzed. The levels of aggressive virulence factors in the rhizosphere soil microorganisms of DN310 were significantly higher than those in the rhizosphere soil microorganisms of DXY under high nitrogen conditions (Figure 1G).

### 2.2. Effects of High Nitrogen on the Functions of Rhizosphere Soil Microorganisms

**Figure 2:**
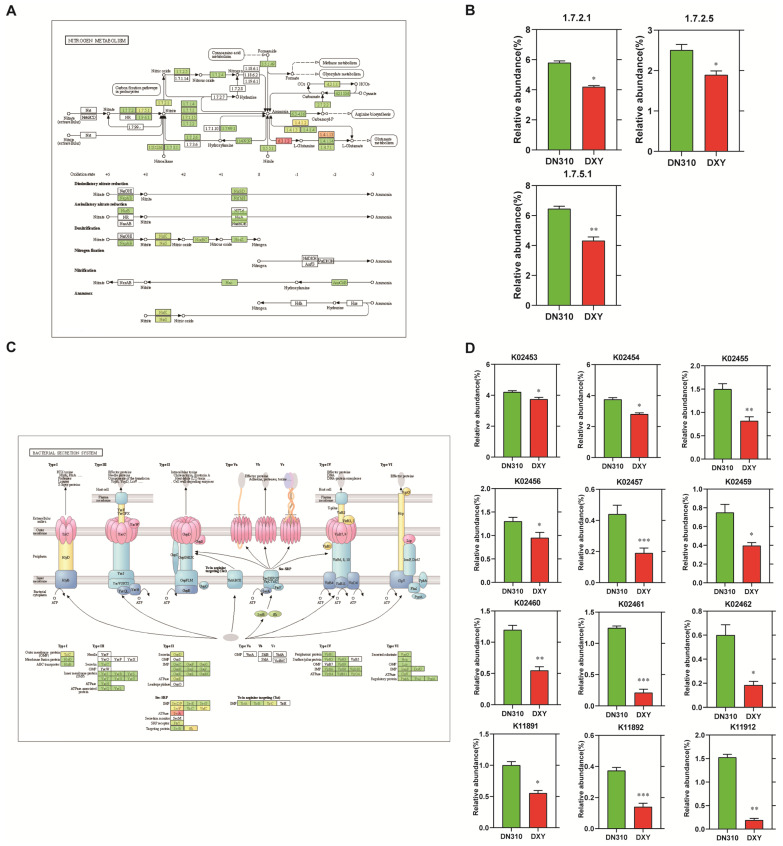
The difference in rhizosphere microbial function in different potato varieties under high nitrogen conditions. (**A**) KEGG N metabolism pathway analysis. The rectangular box represents the enzyme or component in the pathway, and a darker color indicates a higher relative abundance. (**B**) Comparison of relative abundance of enzymes or components in the nitrogen metabolic pathways of DN310 and DXY under high nitrogen conditions. (**C**) Bacterial secretion system metabolic pathways in KEGG database. Rectangular boxes represent the components of each secretory system. (**D**) Comparison of relative abundances of components of bacterial secretion system of DN310 and DXY under high nitrogen conditions. (**B**,**D**) Welch’s test was used to evaluate the difference; * *p* < 0.05, ** *p* < 0.01 and *** *p* < 0.001.

Soil microorganisms can promote nitrogen conversion through nitrification and denitrification. NO_3_^−^ is the main form of nitrogen utilized by plants. The effects of high nitrogen on rhizosphere soil nitrogen dynamics of DXY and DN310 were studied using rhizosphere microbial metagomic sequencing. The levels of key enzymes in nitrogen metabolism were evaluated. Under high nitrogen conditions, the level of enzymes 1.7.5.1 (nitrate reductase), 1.7.2.1 (nitrite reductase), and 1.7.2.5 (nitric oxide reductase) of decreased in rhizosphere microorganisms of DXY compared with those of DN310 (Figure 2A,B). The conversion of nitrate to nitrite and nitrite to nitric oxide (NO) and nitrous oxide (N_2_O) was decreased. In general, under high nitrogen conditions, DXY exhibited higher NO_3_^−^ utilization efficiency and lower emission of NO and N_2_O. The results of metagomic sequencing revealed that the relative abundance of types II and VI secretory system components [K02453 (GspD), K02454 (GspE), K02455 (GspF), K02456 (GspG), K02457 (GspH), K02459 (GspJ), K02460 (GspK), K02461 (GspL), K02462 (GspM), K11891 (IcmF), K11892 (DotU), and 11912 (PpkA)] in the rhizosphere microorganisms was higher in DN310 under high nitrogen conditions (Figure 2C,D). In conclusion, under high nitrogen conditions, the secretory capacity of types II and VI bacteria in rhizosphere soil of DN310 was strong, which could increase the secretion of root microbial toxicity factors. Therefore, DN310 was more susceptible to diseases than DXY.

### 2.3. Effects of High Nitrogen Conditions on Transcriptional Regulation in Different Potato Varieties

**Figure 3:**
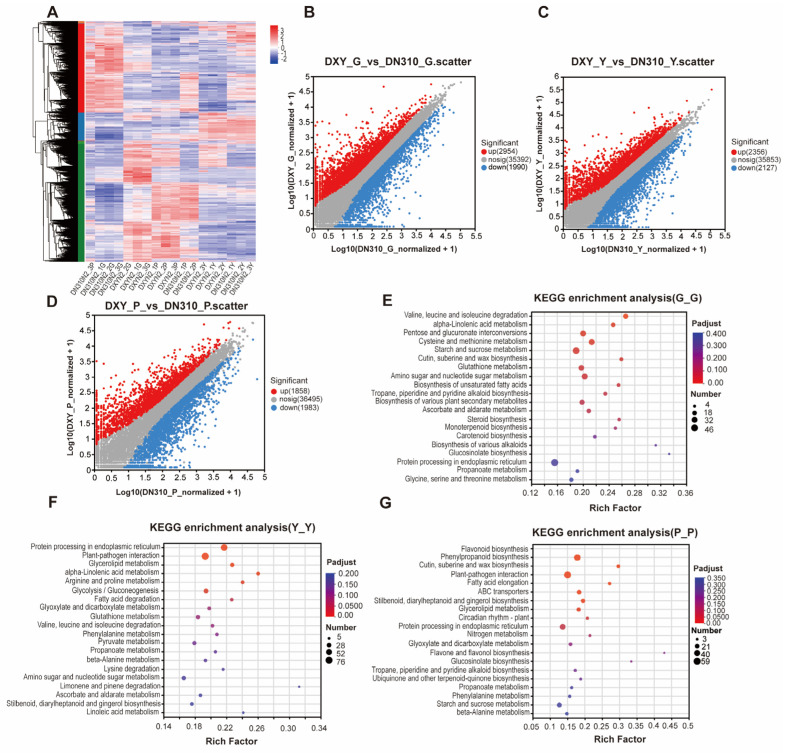
Differences in the transcriptome of different potato varieties under high nitrogen conditions. (**A**) Heatmap of cluster analysis of differentially expressed genes between DN310 and DXY under high nitrogen conditions. Y, G, and R stand for leaves, roots, and stolons, respectively. Red represents higher gene expression, whereas blue represents lower gene expression. (**B**–**D**) Scatter plot of differentially expressed genes of DN310 and DXY under high nitrogen conditions: (**B**) root, (**C**) leaf, and (**D**) stolon, where the horizontal coordinate represents the gene expression of logarithmic DN310, and the vertical coordinate represents the gene expression of logarithmic DXY. Gray dots represent genes that were not differentially expressed, whereas red and blue dots represent genes that were differentially expressed. (**E**–**G**) KEGG pathway enrichment analysis of differentially expressed genes of DN310 and DXY under high nitrogen conditions: (**E**) root, (**F**) leaf, and (**G**) stolon, where the horizontal coordinate represents Rich factor and the vertical coordinate represents KEGG pathway. The greater the Rich factor, the greater the degree of enrichment. The size of the dots indicates how many genes there are in the pathway, and the colors of the dots correspond to different padjust ranges.

To further explore the effects of high nitrogen conditions on potato growth, transcriptome sequencing was performed in DXY and DN310. Many differentially expressed genes were observed in the roots, leaves, and stolons of DXY and DN310 under high nitrogen conditions (Figure 3A). In the root, 4944 genes were differentially expressed in DXY compared with DN310, of which 2954 genes were upregulated and 1990 genes were downregulated (Figure 3B and Supplemental Table S1). In the leaves, 4483 genes (2356 up- and 2127 downregulated, respectively) were differentially expressed in DXY (Figure 3C and Supplemental Table S2). In the stolons, 3841 genes (1858 up- and 1983 downregulated, respectively) were differentially expressed (Figure 3D and Supplemental Table S3). The differentially expressed genes analyzed via KEGG pathway enrichment analysis revealed different patterns in different organs. Specifically, the differentially expressed genes in the roots were mainly enriched in the degradation of valine, leucine, and isoleucine; linolenic acid metabolism; and other pathways (Figure 3E). Differentially expressed genes in the leaves were mainly enriched in the pathways of protein processing, plant–pathogen interaction, and glycolloid metabolism in the endoplasmic reticulum (Figure 3F). Differentially expressed genes in the stolons were mainly enriched in the pathways of flavonoid biosynthesis and phenylpropanoid biosynthesis (Figure 3G).

### 2.4. Effects of High Nitrogen Conditions on the Metabolites of Different Potato Varieties

**Figure 4:**
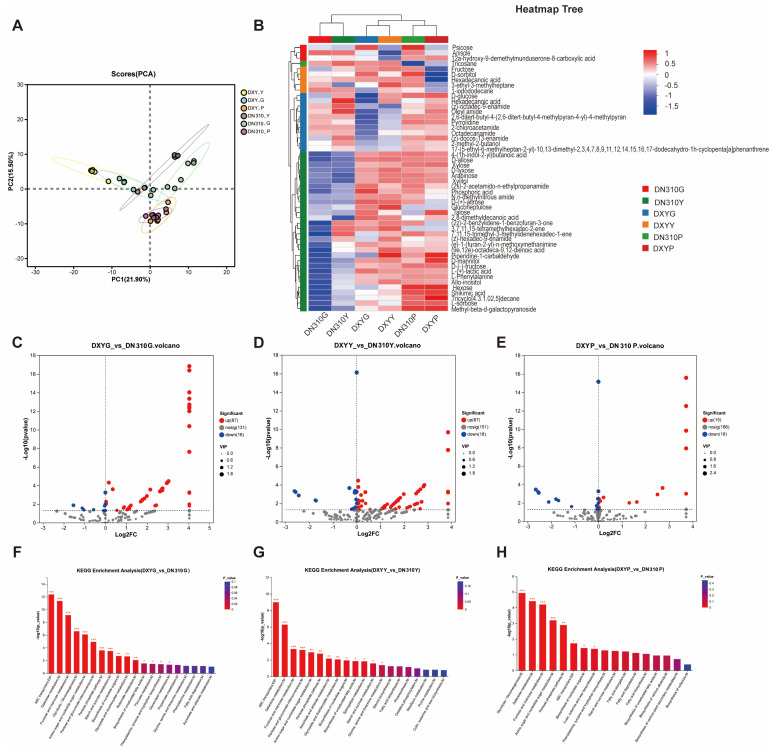
Differences in the metabolites of different potato varieties under high nitrogen conditions. (**A**) Principal component analysis of metabolome sequencing samples. (**B**) Heatmap of cluster analysis of metabolites of DN310 and DXY under high nitrogen conditions. G, Y, and P represent roots, leaves, and stolons, respectively. The colors in the figure represent relative expressions of metabolites. (**C**–**E**) Differential metabolite analysis of (**C**) roots, (**D**) leaves, and (**E**) stolons of different potato varieties under high nitrogen conditions. The horizontal coordinate represents the multiple change of metabolites between DN310 and DXY; the vertical coordinate represents the significant metabolic difference between the two groups, and the red and blue dots represent the differential expression of metabolites between the two groups. (**F**–**H**) KEGG pathway enrichment analysis of differentially expressed metabolites in the (**F**) roots, (**G**) leaves, and (**H**) stolons of different potato varieties under high nitrogen conditions; the horizontal coordinate is the KEGG pathway, and the vertical coordinate is the significance (*p*-value) of enrichment. The smaller the *p*-value, the greater the significance.

Metabolome sequencing was used to study the effects of high nitrogen conditions on metabolites of different potato varieties. First, the principal component analysis of the sequencing results revealed that the samples were repeatedly biologically clustered. The samples under different treatments were clustered into independent clusters at the metabolic level, indicating the specificity of metabolites between samples (Figure 4A). Notably, high nitrogen conditions significantly affected the metabolites of DXY and DN310 (Figure 4B). Under high nitrogen conditions, 103 differentially expressed metabolites were observed in DXY roots compared with DN310 roots, of which 87 were upregulated and 16 were downregulated (Figure 4C and Supplemental Table S4). Similarly, 105 differentially expressed metabolites were identified in DXY leaves, of which 87 were upregulated and 18 were downregulated (Figure 4D and Supplemental Table S5). In total, 31 differentially expressed metabolites were found in DXY stolons, of which 15 were upregulated and 16 were downregulated (Figure 4E and Supplemental Table S6). KEGG pathway enrichment analysis of different metabolites indicated different metabolic pathway enrichment in different organs. Differential metabolites in the roots were mainly enriched in ABC transporters, galactose metabolism, fructose and mannose metabolism, and glycolysis/gluconogenesis pathways (Figure 4F). In the leaves, differential metabolites were mainly enriched in ABC transporters, galactose metabolism, fructose and mannose metabolism, and mutual conversion of pentose and glucuronate (Figure 4G). In the stolons, differential metabolites were mainly enriched in glycolysis/gluconogenesis, galactose metabolism, fructose and mannose metabolism, and metabolism of amino and nucleotide sugars (Figure 4H).

### 2.5. Identification of the Key Genes of Nitrogen Fertilizer Response Using Combined Transcriptome and Metabolome Analyses

**Figure 5:**
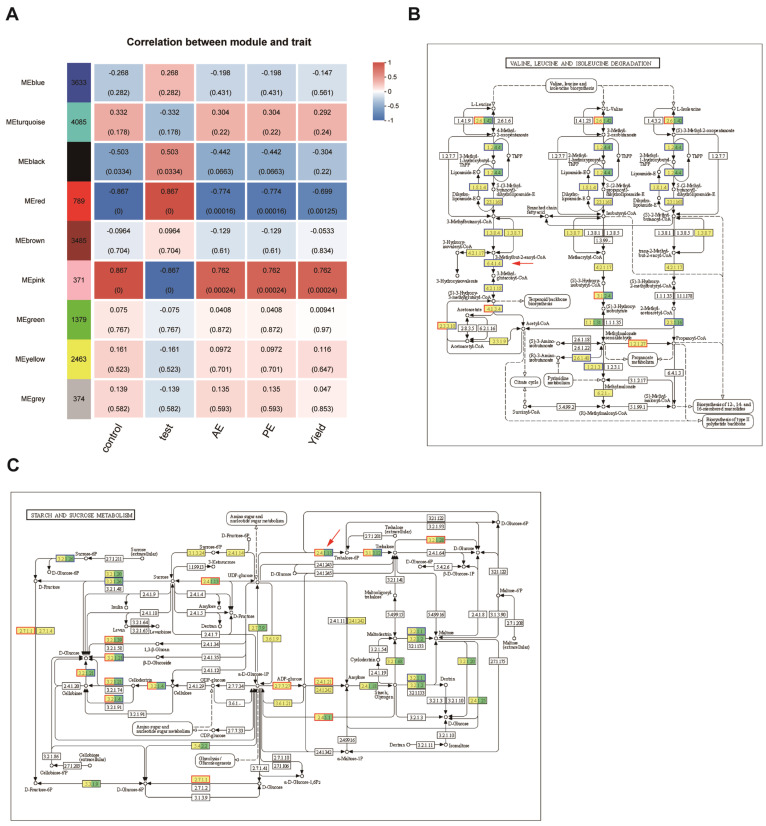
Combined transcriptome and metabolome analyses of key genes related to nitrogen efficiency in potato. (**A**) WGCNA was used to analyze the relationship between nitrogen response modules and phenotypes. The vertical coordinate represented different gene modules. The horizontal coordinate represented the phenotypes of DN310 and DXY, where AE represented the nitrogen agronomic efficiency; PE represented the nitrogen physiological efficiency; Yield represented the yield; the darker color represented the higher correlation degree between the component and phenotype, and the number in the grid represented the correlation coefficient. The numbers in parentheses indicate the level of significance. (**B**) Degradation pathways of valine, leucine, and isoleucine. (**C**) Starch and sucrose metabolic pathways. The red arrows in (**B**,**C**) indicated the proteins affected by the genes.

The WGCNA analysis was conducted using nitrogen agronomic efficiency, nitrogen physiological efficiency, and tuber yield as parameters. Nine modules were obtained, among which the MEred module had the highest correlation with nitrogen agronomic efficiency and nitrogen physiological efficiency module. Therefore, the MEred module was the focus of the study, which contained 789 genes (Figure 5A). Previous transcriptome and metabolome analyses demonstrated that high nitrogen conditions had significant effects on amino acid metabolism and glucose metabolism in both DXY and DN310 (Figure 3 and Figure 4). It was observed that two genes, *PGSC0003DMG400025888* and *PGSC0003DMG400017276*, contained in the MEred module were also closely related to amino acid metabolism and sugar metabolism. The *PGSC0003DMG400025888* gene was mainly involved in the synthesis of 3-methyl-pentenyl-coenzyme A in the degradation pathway of valine, leucine, and isoleucine (Figure 5B). The *PGSC0003DMG400017276* gene was mainly involved in the synthesis of trehalose 6-phosphate in the metabolic pathway of starch and sucrose (Figure 5C).

## 3. Discussion

Nitrogen plays an important role in the nutritional development and production of potato. The potato root is small and shallow and has low nitrogen utilization efficiency. Therefore, to obtain high yield, a lot of nitrogen is required [20]. However, overapplication of nitrogen fertilizer can cause the conversion of unabsorbed nitrogen into nitrous oxide (N_2_O), leading to global warming and environmental pollution [21]. Breeding varieties with high nitrogen efficiency can maintain high yield and reduce environmental pollution with less nitrogen application. In this study, metagenomic sequencing of rhizosphere microorganisms of two potato varieties with different nitrogen efficiency revealed the mechanism of differences in nitrogen use efficiency and the trend of greenhouse gas emissions from the perspective of microorganisms. This provided a reference for breeding potato varieties with high nitrogen efficiency. Under high nitrogen conditions, significant differences were observed in microbial populations in rhizosphere soil between DN310 and DXY, which resulted in differences in the relative abundance of key enzymes of nitrogen metabolism pathway in rhizosphere soil. Under high nitrogen conditions, the amount of nitrate reductase and nitric oxide reductase in rhizosphere microorganisms of DN310 was higher than that in rhizosphere microorganisms of DXY. Thus, the activities of nitrate reductase and nitric oxide reductase were higher in rhizosphere soil of DN310 than in that of DXY, resulting in more conversion of nitrate into nitrite in the soil of DN310 root system. More nitrites are converted into greenhouse gases and emitted into air. Nitrate is the main form of nitrogen fertilizer absorption and utilization by plants. The rhizosphere nitrate of DN310 is converted into greenhouse gases, reducing the available nitrogen in the soil and resulting in low nitrogen utilization efficiency of DN310. Therefore, the results revealed that DXY is a nitrogen-efficient and environmentally friendly potato variety compared to DN310.

Excessive nitrogen fertilizer is often used in potato production to increase yield [22]. However, excessive nitrogen application can increase susceptibility of plants to diseases by affecting plant growth, physiological metabolism, and nutrient composition [23,24,25]. Macrotome sequencing analysis of potato rhizosphere microorganisms revealed that excessive application of nitrogen fertilizer increased the transcript levels of enzymes related to the soil type II and type VI secretory systems, further producing more aggressive virulence factors that affect crop growth [26]. Therefore, in potato production, it is necessary to select nitrogen-efficient varieties that are not susceptible to diseases. In this study, rhizosphere microbial metagenomic sequencing of two potato varieties with different nitrogen efficiency was performed. Under high nitrogen conditions, compared with DXY, the secretion system of type II and type VI bacteria in rhizosphere soil of DN310 had stronger secretion capacity, and more virulence factors were secreted, potentially increasing disease susceptibility.

With the development of sequencing technology, the application of combined transcriptome and metabolome analyses has become increasingly common. Xin studied the transcriptome and metabolome changes of rice under different nitrogen treatment conditions and reported some genes and metabolites related to carbon and nitrogen metabolism [27]. Through the combined analysis, Duan identified some key genes and metabolites involved in nitrogen fertilizer response, which play an important role in nitrogen response [28]. In this study, two potato varieties with different nitrogen efficiency were jointly analyzed. The differentially expressed genes and metabolites were mainly enriched in amino acid metabolism and glucose metabolism pathways under high nitrogen conditions. In addition, WGCNA confirmed the phenotypic significance of MEred gene module. Genes in this module were analyzed in conjunction with differentially expressed genes in amino acid metabolism and glucose metabolism pathways. Finally, two genes were identified, both belonging to the MEred module and differentially expressed genes in amino acid metabolism and glucose metabolism pathways. Although two genes that may be involved in improving potato nitrogen use efficiency were identified, further transgenic experiments are needed to confirm the effects of these two genes on potato nitrogen use efficiency.

## 4. Materials and Methods

### 4.1. Plant Materials and Growth Conditions

In this study, two potato varieties, namely, Dongnong310 (DN310) and Daxiyang (DXY), with different nitrogen utilization efficiency, were used as experimental materials, and they had similar growth periods of approximately 100 days. For the rhizosphere metagenomic sequencing experiment, approximately 50 g seed tubers were planted at Northeast Agricultural University in Harbin, China. The test site is 128 m above sea level; the average annual rainfall is 550 mm; the soil type is chernozem; and the soil fertility is medium and uniform. Two nitrogen treatments were administered, including N0 (0 kg of urea, 15 kg of superphosphate, and 20 kg of potassium sulfate per 667 m^2^) and N1 (30.4 kg of urea, 15 kg of superphosphate, and 20 kg of potassium sulfate per 667 m^2^). Each treatment was repeated 3 times. Each plot for each treatment had 10 rows; the row spacing was 80 cm; the plant spacing was 25 cm; and the length was 5 m. Appropriate field management and pest control were conducted during plant growth. For transcriptome and metabolome sequencing experiments, the plants were grown in pots in a greenhouse under controlled conditions with 16 h of light and 8 h of darkness. Day and night temperatures were maintained at 25 °C and 21 °C, respectively. Coir fiber was used as the medium, and MS (Cat.PM1281, Coolaber, Beijing, China) with 0.495 g/L NH_4_NO_3_ was used as the nutrient solution.

### 4.2. Determination of Nitrogen Use Efficiency

The total nitrogen content of DXY and DN310 was determined at N0 and N1 levels as described previously [29]. Each treatment was repeated three times. After the plant samples were digested using sulfuric acid–hydrogen peroxide, the digestion solution was put into the automatic Kjeldahl nitrogen analyzer (K9860, Haineng, Jinan, China) to determine the nitrogen content. Further, nitrogen use efficiency was calculated according to the following formula:

Nitrogen physiological efficiency (PE; kg/kg) = (Yield in nitrogen-treated area − yield in blank area)/(Nitrogen uptake by plants in nitrogen-treated area − Nitrogen uptake by plants in blank area)

Nitrogen agronomic efficiency (AE; kg/kg) = (Yield in nitrogen application zone − yield in blank zone)/Nitrogen application amount.

### 4.3. Analysis of Rhizosphere Microbial Metagenomic Sequencing

The rhizosphere soil (0.5 g) of two potato varieties grown at N1 level were taken as bioreplicates, with three bioreplicates for each variety. Macrogenomic sequencing of rhizosphere microorganisms was performed as described previously [30]. In brief, the total DNA of soil microorganisms was extracted and processed using ultrasound ultrasonic cell disruptor (M220, Covaris, Shanghai, China)to 350-bp size. Subsequently, library construction and sequencing of these fragments were conducted on the Illumina NovaSeq platform (NovaSeq6000, Illumina, Shanghai, China).

#### Gene Prediction, Microbial Species Annotation, KEGG Functional Annotation, and VFDB Virulence Annotation

According to the method by McNair et al. [31], the Prodigal v2.6.3 splice software (https://github.com/hyattpd/Prodigal (accessed on 2 September 2024)) was used to predict open reading frame from the sequencing results. The genes with more than 100 bp are translated into proteins. According to the method proposed by Buchfink et al. [32], DIAMOND software (http://ab.inf.uni-tuebingen.de/software/diamond/ (accessed on 15 September 2024)) was used to compare the non-redundant genes with the core databases such as NR, KEGG, and VFDB to obtain the species, function, and toxicity annotations of microorganisms. The comparison type was blastp, and the E-value was ≤ 1 × 10^−5^.

### 4.4. Transcriptome Sequencing

At the tuber expansion stage, 0.2 g each of roots, leaves, and stolons of two potato varieties were collected, with three biological replicates for each organ. RNA was extracted using a kit (Cat.LS1040, Promega, Beijing, China). RNA concentrations were measured using Nanodrop 2000. RNA integrity was assessed using 1% agarose gel electrophoresis. After RNA fragmentation, reverse transcription was performed. cDNA tail was added; libraries were constructed, and transcriptome sequencing was performed using Illumina on the Nova BioSeq platform.

### 4.5. Metabolome Sequencing

According to the method by Xu et al. [33], metabolome sequencing was performed using the root, leaf and stolon samples of different potato varieties. After thawing the samples on ice, the metabolites were extracted with methanol and incubated at −20 °C for 1 h. The supernatant after quality control was subjected to UHPLC analysis. The mobile phase of the UHPLC system consisted of 25 mM NH_4_OAc.

### 4.6. Differentially Expressed Genes and Weighted Gene Co-Expression Network Analysis

According to the method described by Love et al. [34], gene expression levels were estimated per million mapping fragments per kilobase transcript fragment, and a negative binomial distribution-based model was used to identify differentially expressed genes between two potato varieties. Weighted gene co-expression network analysis (WGCNA) was performed using the WGCNA package in R [35]. The soft power was 7; minModuleSize was 30; minKMEtoStay was 0.3, and mergeCutHeight was 0.25.

## 5. Conclusions

**Figure 6:**
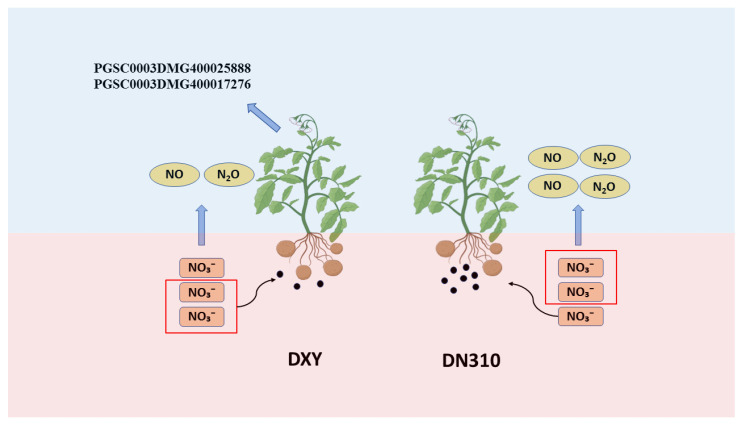
Schematic diagram of the study. The black dots represent virulence factors, and the number of shape components represents the amount of substance produced or transformed.

In this study, metagenomic sequencing technology was used to study the differences in rhizosphere microbial populations of two potato varieties with different nitrogen efficiency under high nitrogen conditions. The mechanisms underlying the differences in nitrogen utilization, rhizosphere greenhouse gas emissions, and disease susceptibility were revealed from the perspective of microorganisms. Two key genes involved in nitrogen fertilizer response were identified using combined transcriptome and metabolome sequencing analyses, which provided a new idea for efficient breeding of potato varieties with high nitrogen efficiency. The main conclusions of this study (Figure 6) are as follows: (1) Compared with DN310, DXY was a nitrogen efficient variety with higher nitrogen use efficiency. (2) Under high nitrogen conditions, the relative abundance of nitrate reductase and nitrite reductase genes in rhizosphere microorganisms of DXY was lower than that of DN310; the NO_3_^−^ content directly available to plants increased, thus improving the nitrogen utilization efficiency. (3) Under high nitrogen conditions, compared with DN310, the rhizosphere microorganisms of DXY exhibited a relatively low abundance of nitric oxide reductase genes and emitted less NO, thus causing less environmental pollution. (4) Under high nitrogen conditions, compared with DN310, the relative abundance of key enzymes of type II and type VI secretory systems in rhizosphere microorganisms of DXY was lower, resulting in fewer virulence factors secreted by microorganisms. Therefore, DXY may have a weaker disease susceptibility. (5) Two nitrogen effector genes, namely, *PGSC0003DMG400025888* and *PGSC0003DMG400017276*, were identified using combined transcriptome and metabolome analyses, which provided valuable insights into breeding potatoes with high nitrogen efficiency.

## Data Availability

The data that support the findings of this study are available from the corresponding author upon reasonable request.

## Supplementary Materials

The following supporting information can be downloaded at: https://www.mdpi.com/article/10.3390/plants14050633/s1, Table S1: Supplemental Table S1.csv, Table S2: Supplemental Table S2.csv, Table S3: Supplemental Table S3.csv, Table S4: Supplemental Table S4.csv, Table S5: Supplemental Table S5.csv, Table S6: Supplemental Table S6.csv.

## Abbreviations

The following abbreviations are used in this manuscript:

N	Nitrogen
NUE	N use efficiency
PTI	Pattern-triggered immunity
ETI	Effector-triggered immunity
DXY	Daxiyang
DN310	Dongnong310
PE	Nitrogen physiological efficiency
AE	Nitrogen agronomic efficiency
